# Experiences of specially trained personnel of group education for patients with type 2 diabetes—A lifeworld approach

**DOI:** 10.1002/nop2.248

**Published:** 2019-02-21

**Authors:** Susanne Andersson, Mia Berglund, Caroline Vestman, Anna Kjellsdotter

**Affiliations:** ^1^ School of Health and Education University of Skövde Skövde Sweden; ^2^ Primary Health Care Center Gullspång Sweden; ^3^ Research and Development Centre Skaraborg Hospital Skövde Skövde Sweden

## Abstract

**Aim:**

To describe how the group education process for people with type 2 diabetes is experienced by diabetes nurses and dietitians who support the patients’ learning, in a primary care setting.

**Design:**

The project took place at two primary care settings in the south of Sweden.

**Methods:**

Data collected from focus‐group interviews and reflection notes were subjected to phenomenological analysis.

**Results:**

The specially trained personnel experienced that group education made it possible for the patients to learn through reflection concerning their own and others’ experiences. Furthermore, group education entailed increased knowledge for the trained personnel. When the patients were challenged to make changes in their lives with the illness, the personnel experienced that both patients and personnel supported each other. The study concludes that the trained personnel person‐centred approach, with help of the didactic model, get tools to support patients learning.

## 1 INTRODUCTION

Patients’ learning and education are important when developing a person‐centred care approach (Ekman et al., 2011) that increases the patient's ability to influence and participate, that is, be involved in their own care (Andersson, Svanström, Ek, Rosén, & Berglund, 2015; Friberg, Berglund, Kronberg, & Lecksell, 2015). In health care, the focus has long been on providing information and advice, not on supporting patients’ learning processes (Berglund & Källerwald, 2012). The didactic model, “To take charge in life with long‐term illness” (Table 1), can be used to increase patients’ ability to achieve learning and well‐being in the case of diabetes mellitus type 2 (T2D) (Andersson et al., 2015; Berglund, 2011).

**Table 1:** The thesis in the didactic model “The challenge—to take charge in life with long‐term illness.” A tactful, challenging approach is profound in the model

Confronting one's life situation and challenging oneself to make changes
Positioning oneself at a distance when creating a new whole
Developing self‐consciousness and taking responsibility from “one” to “I”
Making learning visible with the aim of achieving development and balance in life

## 2 BACKGROUND

By 2017, it was estimated by the International Diabetes Federation (IDF) that 8.8% (58 million) of Europe's population aged 20–79 would have diabetes including 22 million undiagnosed cases. The figure is expected to increase to 66.7 million people with diabetes by 2045; the increase in T2D is primarily due to the ageing population in this region (International Diabetes Federation, 2017). Further, the healthcare expenditure spent on people with diabetes in Europe is high and more than 9% of all mortality is attributed to diabetes. The disease is a progressive disease, and the overall goal is to prevent, delay and control complications by effective measures (World Health Organization, 2017). According to the Swedish National Diabetes Register (NDR), the 2015 annual report (NDR, 2015) shows that the average age of persons cared for in primary care setting with T2D is 68.2 years of age; furthermore, they have had diabetes for 9.2 years which makes caring for diabetes a major issue.

Most patients with T2D in Sweden have their care contact in the primary care setting (NDR, 2015). Primary care gives people with diabetes counselling, support, treatment and patient education, individually and/or in groups to increase self‐management. Group‐based patient education means that the patients are offered the opportunity to participate in education with other people with diabetes and it consists of structured programmes which assume active participation. The programmes are usually based on educational theories, models or knowledge of adult learning, as in this study the didactic model, “To take charge in life with long‐term illness” (Table 1). The model used is based on the patient's understanding and challenges it on both a cognitive and an existential level as described in previous research (Andersson et al., 2015). The groups can be led by various health professionals who have subject and educational skills, in this study diabetes nurses and dieticians (SBU, 2009). The National Board of Health and Welfare (2018) recommends that a structured group‐based patient education for people with T2D is offered. Group‐based patient education given with the above conditions gives a significant reduction of Hba1c (Scain, Friedman, & Gross, 2009).

Health professionals in primary care prioritize and take responsibility for the patient's learning in their daily work and have a higher degree of pedagogy at university level, compared with nurses in other care‐related environments (Bergh, Persson, Karlsson, & Friberg, 2014). The specially trained diabetes nurse in primary care may experience ambivalence about the ability to practise patient‐centred care, concerning the professional role and a position of withdrawn expertise as a nurse. Instead of providing information, the alternative role was to listen, discuss and interact with the patient (Boström, Isaksson, Lundman, Lehuluante, & Hörnsten, 2014). Patient‐centred interaction during group sessions can strengthen the healthcare professionals in their professional role (Boström, Isaksson, Lundman, Graneheim, & Hörnsten, 2014) and can significantly decrease Hba1c at 12‐month follow‐up (Jutterström, Hörnsten, Sandström, Stenlund, & Isaksson, 2016).

To integrate a long‐term illness, such as T2D, into a life context requires considerable efforts (Whittemore & Dixon, 2008), and the integration is one important task for the specially trained diabetes nurse to support. Illness integration and self‐management can be a parallel process that ends up in a turning point (Hörnsten, Jutterström, Audulv, & Lundman, 2011). The turning point occurs when the illness is experienced as more severe and may affect the self‐management and make the patient more engaged in the learning process (Jutterström, Isaksson, Sandström, & Hörnsten, 2012). The duration of illness is not of importance for the patient's learning process, which emphasizes a person‐centred care to meet the different and changing needs when living with lifelong illness such as T2D (Kneck, Fagerberg, Eriksson, & Lundman, 2014).

Learning as a concept is double‐sided, either teaching by showing or practicing on someone else, or learning something by the person himself being active in acquiring knowledge through experience and insight (Berglund & Källerwald, 2012). A way of reflective learning as a person is transforming experiences to new knowledge and skills by thinking, doing and feeling (Jarvis, 2006). Genuine learning should be supported at an existential level and involves the patients’ thoughts, feelings, actions and reflections of their experiences (Berglund, 2014; Johansson, Almerud Österberg, Leksell, & Berglund, 2015), which requires openness and support from the health professionals (Friberg et al., 2015). The support provided challenges and encourages the patients to reflect about their own goals, expectations and needs (Johansson, Almerud Österberg, Leksell, & Berglund, 2018) and leads to a greater understanding of the patients’ life situation with the illness (Andersson et al., 2015). The patient needs to be confronted with their life situation and challenges for changes to realize that the patient is the one who takes charge, instead of the disease controlling the patient (Berglund, 2011). Subsequently, learning can be defined as the person with T2D accepting the illness, with a new understanding of themselves as a person (Johansson et al., 2018).

Supporting patients’ learning processes increases the patient degree of self‐management and involvement in their own care, on good terms, which is essential for a person‐centred care. T2D is a major, increasing health problem in Sweden, which makes caring for patients with T2D in a primary healthcare setting a great burden. Persons with T2D need the health professionals’ support. Individual counselling is the most common form of education in diabetes care, although group education is highly recommended. In the literature, there is little describing how group education of persons with T2D is experienced by diabetes nurses and dietitians, particularly in terms of supporting learning based on a didactic model, in a primary care setting.

## 3 THE STUDY

### 3.1 Design

#### 3.1.1 Setting and project

The project “To take charge in life with T2D—a model for group education in primary care” took place in two primary care settings in the south of Sweden and involved two registered nurses and three dietitians who had special education and long experience (3–17 years) of counselling and diabetes care. The health professionals who facilitate the education sessions had graduated from a university course about the didactic model based on a lifeworld approach: The challenge—to take charge in life with long‐term illness. The model aims to support patients’ learning on an existential level (Andersson et al., 2015). Learning from a lifeworld perspective can be understood as an altered understanding that requires and is created through reflection and dialogues. Creating a reflective dialogue with patients is central to this model. A dialogue to challenge the person with T2D into reconciliation with the actual situation is therefore recommended. The health professionals learn to apply a tactful and challenging approach based on the patient's life situation (Berglund, 2011, 2014). The participants, who were recruited from two primary care units, participated in five group meetings lasting 2 hr every other week based on various themes (Table 2).

**Table 2:** The themes reflected upon during the group meetings

Meeting 1	My idea of the illness T2D
Meeting 2	Who am I?—Who am I with T2D?
Meeting 3	Then—Now—Future
Meeting 4	Obstacles—Opportunities, Strengths—Weaknesses
Meeting 5	My goals, challenges—my own learning process

T2D, diabetes mellitus type 2.

### 3.2 METHOD

#### 3.2.1 Data collection

Three focus‐group interviews with the nurses and dietitians were conducted before, during and after the group‐based education programme, in a separate room at the University by two researchers (SA, MB). The focus‐group interviews were carried out as a dialogue, to enable the participant to reflect together about the phenomenon of interest (Kvale, 2009). At the first group interview, two nurses and three dietitians participated. After the first interview, one dietitian chose to leave the study due to a lack of diabetes nurses who could participate in group education. In the second and third interviews, four informants participated. Each interview lasted between 27–81 min. The interviewees used open questions and follow‐up questions. During the interviews, the researchers took notes. They made short summaries to clarify that they all understood each other correctly. The informants responded in the order they desired. Sometimes, the informants described with gestures and without words. The interviewees were responsive to this, and verbal clarification was made. During the first interview, the focus was on the previous experience of the health professional in teaching patients with T2D and their expectations and concerns about the new way of teaching. The second interview focused on the health professionals’ experiences about the first group meeting with the patients. The last interview focused on the health professional experiences after the last group meeting with the patients. The interviews were digitally recorded and transcribed verbatim. After each training session with the patients, written reflections were collected from the participants which gave an opportunity to write down their unique experiences in a reflection book included in the educational material (Dahlberg, Dahlberg, & Nyström, 2008). All the participants spoke Swedish.

#### 3.2.2 Analysis

The phenomenon of this study is diabetes nurses’ and dietitians’ experience of group education of persons with T2D, before, during and after implementation. Using the reflective lifeworld research (RLR), a research approach developed by Dahlberg et al. (2008) based on Giorgi's (2009) phenomenological approach, the phenomenon is explored and illuminated. According to phenomenological philosophy, people and their existence can never be understood, without being considered a living whole. The reflective lifeworld approach is focusing on how the world with its everyday phenomena is lived, experienced, accomplished and described by humans aiming to clarify and describe lived experiences to increase knowledge of an individual personal experience.

Overall, all text is seen as data, as one whole piece of text. In the first phase of the analysis, the text was read in an open manner to get acquainted with the data. All data were then divided into meaning units, a sequence of the text that has a meaning of its own. Each meaning is reflected against the background of the whole. The next phase involves building groups of meanings, as clusters. Finally, the essence is formed, which is described by Dahlberg (2006) as an abstraction and synthesis of a phenomenon's unique structure of meanings. The essence with structure and nuances of the phenomenon can be understood as a new whole.

In this paper, the essence is presented in the results. The phenomenon under study is further enlightened by the five constituents: Creating prerequisites for safety within the group, learning through reflection, taking a listening and leading role, challenges versus confidence and using tools to stimulate reflection. The results are illustrated with quotations from the interviews.

#### 3.2.3 Ethics

A written informed consent was obtained from all those involved, the participants, course participants and responsible manager, and the study confirms the principles outlined in the Declaration of Helsinki (WMA, 2008). The study was approved by the Central Ethical Review Board, University of Gothenburg. (Dnr: 442–15).

## 4 RESULTS

The phenomenon of health professionals experience to supporting learning, based on the didactic model '*To take charge in life with T2D—a model for group education in primary care*', is a challenge for the health professionals. For health professional, it is challenging to wait for the participants’ reflections, listening to them with an open mind and staying in the background, not deciding the coming subject. The health professionals challenge the participants to think and try to find out the answers themselves not always answering their questions. In a tactful way, they ask the participants to reflect on their own experiences of living with T2D. The didactic model gives the health professionals the ability to meet the unique person in their life with T2D enabling a deep relation to the participants by creating safety, support and trust. A tactful and open approach is required to get all the participants in the group to share their experiences from their daily life with diabetes. Tools such as writing, drawing pictures and selecting amongst other pictures are used to support reflection. The supportive and permissive climate that the health professionals experience gives an interchange of knowledge exchange controlled by the participants. The health professionals think that the model, with its approach, creates possibilities to support a deep learning based on reflections and sharing.

### 4.1 Creating prerequisites for safety within the group

The health professional experiences of group education in T2D indicate the importance of creating conditions within the group so that the participants feel safe and free to share their experiences. The first round of conversation (the participants share their experiences, one by one) in the group is therefore particularly important and controls the coming content. The round enables reconnecting to subjects brought up later during the meeting. The responsibility for the group activity rests on the health professionals, which can be challenging. The challenge consists of meeting the more silent participants and knowing if they reflect and learn. Even though other participants share their experiences, it does not necessarily inspire the silent participant to do the same. The health professionals feel that it is important for the participants to have the space to share their experiences to the extent they wish. The repeated group meetings create deeper relationships with the participants. A health professional describes the difference between meeting participants in a group, compared with individual counselling:

And you will hear a little more about how they are thinking and reasoning and see things in a completely different way. In this way you get a greater understanding of the person than you get at an individual short meeting.

The health professionals believe that the rounds can convey safety but may also be perceived as pressing in those cases where the patient has not made any major changes since the last group meeting. The participants have signed a contract of confidentiality in the group. The contract is a prerequisite for sharing experiences and contributes to the security of the group.

### 4.2 Learning through reflection

The participants learn about their illness and how to live life with T2D by reflecting about their own and others’ experiences. The health professionals are concerned that medical evidence will not be affected. Consequently, a lot of questions are raised and discussed. The health professionals feel confident in answering the participants’ questions and that they have the knowledge required:

It is nice to have done this in a different way compared to before. We have the material, we can talk about it. Eh, so it's nothing to worry about

During the group‐based education, the health professional experiences that the participants discuss and reflect over raised questions in a deeper manner, compared with their earlier experiences. They often find the answer together within the group. The group requests facts and discusses issues such as food, blood sugar levels and diabetes in relation to their habits and lifestyle. Through self‐control of blood glucose levels, discussions and reflections are created at the group meetings:

It will be something completely different. And especially when they share it. Then the whole group has made that experience in some way

The group discussions clarify the participants’ views on what illness means to them as a person. They all have T2D, but they are still unique as a person. The group education raises insight and awareness which improves the preparedness and ability to take responsibility for the outcome:

Then there are actually several participants who draw pictures of themselves with a fat stomach before diabetes. Flat stomach after diabetes

The health professional notes that the adjustments in lifestyle lead to improved blood glucose levels and improved weight reduction, achieved during the ongoing group education.

### 4.3 Taking a listening and leading role

The health professional creates the conditions for all participants to share their experiences by the rounds. It is crucial to be prepared, to listen to the reflections in the group and to take notes to support learning. One way to stimulate the group conversation is to address an open question that gives open answers. The participants then take charge of “what and when” to discuss during the group meeting. The introduction is important as it determines what the continuation will be like. It is good to start the group meeting with a question initiated by the participants as it creates reflections in the group. Participants are given an opportunity to share their experiences of living with T2D with someone else in the same situation without sharing their daily life. This creates good conditions for open reflections:

You can really talk in a way you may not do at home or with anyone and it is not anyone who listens

The group education based on this model gives participants the permission to reflect over thoughts and feelings about their illness by exchanging experiences with each other. The health professional does not always give the answer; instead, they challenge the participants to find their own answers.

### 4.4 Challenges versus confidence

The health professionals express an uncertainty about how participants will experience group education. They describe how the health professionals feel safe about having a theoretical basis in the model. However, they also argue that the participants will experience the model as unclear and therefore feel insecure. Consequently, they see it as a challenge to venture out onto a new and uncertain path. On the other hand, they feel a degree of security since they have previously completed group tuition, which contributes to the calm they feel about the task:

It will be ok, as it has every time so far

Instead, the group contributes to the health professionals’ own learning. The participants share their thoughts and feelings of importance; these are things that are unknown to the health professionals. The didactic model includes challenging moments for the health professionals, such as waiting for the participants’ reflections, listening to the participants with an open mind, not responding too quickly and staying in the background with the aim of allowing the participants to decide the content. Before the start of the group education, the health professionals were concerned about being two members of staff, instead of one. This proved to have the opposite effect as there is an interaction between the dietitian and the diabetic nurse which makes them feel secure. The education is challenging even for the health professionals, and together, they can reflect and support each other and gain a broader range of skills. Sometimes, they want to go back to the old way of teaching. Therefore, the group meeting and the ongoing supervision make the health professionals feel even more secure and confident.

### 4.5 Using tools to stimulate reflection

Various tools are used in addition to blood glucose measurement to stimulate reflection. The participants are asked to draw something, based on a specific thought. It makes it easier for those who find it hard to express their thoughts and feelings on their minds. Drawing and writing are used as a tool that stimulates reflections. The health professionals can see a difficulty for the participants who do not feel comfortable with drawing or writing. Pictures are another tool that worked well which was used to start reflections in the group. Each participant selects a picture to reflect around:

And they chose (the picture) with care. So it was more of a concrete thing that gave us some fun

The supervision material helps the health professionals to reflect on what has happened between the meetings. It seems to be a challenge using the tools at the right time. The participants have voluntarily chosen to write and draw their reflections in the supervision material. The health professionals believe that it is enough to handle one question per meeting and then let the participants control the content with their questions and thoughts. As a health professional, you have support in the material and use it based on what participants ask for. This means that the health professional does not have to prepare for the group education. It turns out that the method of conducting group discussions makes the participants aware that they can do things differently. There is nothing right or wrong. Preparations for the meetings require less preparation for the health professional since they do not lecture. The group meetings do, however, take some time.

## 5 DISCUSSION

The following results are discussed in relation to the didactic model's four principles (Table 1). The study shows that a relationship built on trust and safety between health professionals and participants develops during the group education based on the didactic model, with a lifeworld perspective, compared with earlier experience of more traditional education, based on a medical approach. One of the theses in the didactic model: *“confronting one's life situation and challenging oneself to make changes”* requires a tactful, open, listening and challenging approach (Berglund, 2011). This approach entails that when the health professionals meet the patient, the health professionals must have a genuine will to understand the patient from the patient perspective (Dahlberg, 2006). Therefore, the health professionals’ approach must be person‐centred, which may be experienced as an altered professional role (Boström, Isaksson, Lundman, Graneheim et al., 2014) as well as a challenge to translate into practice (Zoffmann et al., 2016) The approach requires the courage to address questions on an existential level, to stay and listen to the answer (Andersson et al., 2015).

The health professionals received supervision before, during and after group meetings. Supervision is helpful to their own learning process to distance themselves and visualize their use of the model (Andersson et al., 2015). There is a need to be supported and mentored in this role to be able to support the patients to self‐reflection and be responsible for their diabetes (Johansson et al., 2018). As a health professional, it is crucial to see to it that everyone in the group feels secure and free to reflect, exchange reflections and express feelings related to the diabetes; this is supported by Boström, Isaksson, Lundman, Graneheim et al. (2014). The model supports the reflective process amongst the group participants. This study shows that health professionals learn to ask questions that give the participants the possibility to review their life and experiences with the illness. It is necessary to adopt a distant position using a reflective process by: '*positioning oneself at a distance when creating a new whole*' which contributes to the health professionals’ own learning. Therefore, the group discussions increase insight within the group, despite their suffering from the same illness, they are still unique as a person, seen as a person and not as an illness or disease.

The tools used in this model, such as drawing, writing, drawing pictures and seeing other pictures and home assignments, allow participants to reflect together. The participants in this study received home assignments in the form of measuring blood sugar values related to eating and exercising. They reflected on the outcome within the group, and this provided meaningful discussions. The participants “develop self‐consciousness and taking responsibility from “one” to “I” by reflecting about the actual situation in the group, related to the illness and by using “I” instead of “one” during the conversation (Berglund, 2011). The conversation is conducted in the form of rounds and the health professionals must keep still and quiet, which can be perceived as difficult. Deep dialogues with patients, move care to a higher level (Andersson et al., 2015) to support learning instead of providing information. The results of the study support the fact that health professionals should pay attention to how the patient is talking about himself, the disease and if any resistance exists. This is done to support the patient to move on to taking responsibility from “one” to “I.”

The health professionals feel that the model creates a deeper learning in comparison with previous group education. The supportive and permissive climate gives a knowledge exchange controlled by the participants and was supported by the different professional competences. It is important to achieve a interaction between the professionals and patients during the group meetings that are patient‐centred (Boström, Isaksson, Lundman, Graneheim et al., 2014). Being two health professionals was experienced as strengthening their professional roles. Andersson et al. (2015) claim that the aim of the model is to give patients the ability to be in charge of their lives and their treatment, which allows them to show the direction of the care that the health professionals can follow. By putting the patient's choice first, before the counsellor's advice, there is a new way for the health professionals to think (Andersson et al., 2015). The model guides the health professionals to not always answer the patient's questions, but instead, patients are challenged to find the answers themselves. According to Berglund (2011), questions and not answers can support the participant to visualize his learning to reach the feeling of being in progress in life with a long‐term illness. The health professionals note that patients make improvements in their living habits, with reduced blood glucose levels and weight loss. Nurse‐led self‐management in groups improves HbA1c as shown in earlier research (Jutterström et al., 2016). The health professionals experience that patients who choose to participate in group education are more motivated to change lifestyle habits. To “Make the learning visible with the aim of achieving development and balance in life” requires motivation to make lifestyle changes. The integration of a lifelong illness such as T2D is an important task for the nurses and can be seen as a parallel process with self‐management (Hörnsten et al., 2011). Learning involves new knowledge and skills and leads to a change of thinking, acting and feeling (Jarvis, 2006) which is supported by the result of this study. The learning patterns differ between persons, and the strategies differ over a time span (Kneck et al., 2014) and can last for several years, but there is a need for support from health professionals. Prerequisites for the healthcare personnel to take responsibility for the patient's learning are, on the one hand, to have a degree of pedagogy at university level (Bergh et al., 2014) and, on the other, to receive continuous education.

### 5.1 Methodological discussion

The interviewees had many years of experience of conducting interviews in research. The researchers were also trained in the supervision of health professionals in caring and nursing. The group of health professionals may be considered small, but the data contained rich and detailed information. The researchers reflected on their pre‐understanding throughout the study and tried to maintain an open attitude (Dahlberg et al., 2008). Further studies are needed.

## 6 CONCLUSION AND PRACTICAL IMPLICATIONS

The primary application of this approach is to support the learning process for those living with a lifelong illness as diabetes. The result of the study shows that health professionals with help of the didactic model and a lifeworld perspective support patients learning. The health professionals’ learning is facilitated through reflection about patients own and other group members’ experiences. This contributes to the group as well as to the health professionals’ own learning. Consequently, the approach demands the health professional's courage to address questions on an existential level and to have the courage to stay and listen to the answer and is a prerequisite to give a person‐centred care.

## ETHICAL APPROVAL

This study was approved by the Central Ethical Review Board, University of Gothenburg. (Dnr: 442‐15).

## CONFLICT OF INTEREST

The authors have no conflicts of interest to declare.

## AUTHOR CONTRIBUTIONS

SA, MB, and AK: Study Design. SA, MB, CV, and AK: Data collection and analysis. SA, MB, CV, and AK: Manuscript preparation.
